# Silencing miR-21 Sensitizes Non-Small Cell Lung Cancer A549 Cells to Ionizing Radiation through Inhibition of PI3K/Akt

**DOI:** 10.1155/2014/617868

**Published:** 2014-04-07

**Authors:** Yongfu Ma, Hui Xia, Yang Liu, Min Li

**Affiliations:** ^1^Department of Thoracic Surgery, Chinese PLA General Hospital, No. 28 Fuxing Road, Beijing 100853, China; ^2^Department of Thoracic Surgery, The First Affiliated Hospital of Chinese PLA General Hospital, No. 51 Fucheng Road, Beijing 100048, China

## Abstract

We investigated the role of microRNA-21 (miR-21) in radiotherapy resistance of non-small cell lung cancers (NSCLC) and the underlying molecular mechanism. A549 cells were transfected with anti-miR-21 or the negative control oligonucleotides and real-time PCR was applied to detect miR-21 expression level. After ionizing radiation (IR), the survival fractions, proliferation, apoptosis, and expression of phosphorylated-Akt of A549 cells were determined by clonogenic survival analysis, MTT assay, flow cytometry, and Western blotting. Downregulation of miR-21 in radioresistant NSCLC A549 cells inhibited the colony-forming ability and proliferation of A549 cells after IR. Moreover, silencing miR-21 enhanced apoptosis of A549 cells induced by IR accompanied by decreased phosphorylated-Akt protein level. However, PI3K activator IGF-1 reversed suppression of phosphorylated-Akt protein level and promotion of apoptosis of A549 cells after IR caused by miR-21 knockdown. Silencing miR-21 in radioresistant NSCLC A549 cells sensitized them to IR by inhibiting cell proliferation and enhancing cell apoptosis through inhibition of PI3K/Akt signaling pathway. This might help in sensitization of NSCLC to radiotherapy.

## 1. Introduction

Lung cancer is the leading cause of cancer-related deaths worldwide [1], whereas non-small cell lung cancer (NSCLC) represents the most frequent type of lung cancer [2]. NSCLC accounts for approximately 80% of all lung cancer cases and has a 5-year overall survival rate of less than 15% [3, 4]. Approximately 40% of patients diagnosed with NSCLC have unresectable stage III disease or medically inoperable disease [5].

Radiation therapy has been regarded as the main treatment strategy for NSCLC for a long time. However, radioresistance is the key issue limiting the effects of radiotherapy [2, 6]. It is possibly due to tumor heterogeneity in terms of cell of origin, pathology, etiology, and molecular/genetic pathogenesis [7]. NSCLC cells are often resistant to radiotherapy [8], which in turn induces the local recurrence of NSCLC [9, 10]. Therefore, the development of novel approaches for the treatment of NSCLC, including targeted gene treatment as a radiosensitizer to treat this lethal disease, is urgently needed to enhance the survival rate in patients.

microRNAs (miRNAs) [11] are a class of short noncoding RNAs that function as a regulation for gene expression via targeting mRNA for degradation or inhibition of translation [12]. miRNAs are new factors implicated in regulating the expression of genes involved in tumorigenic processes, such as inflammation, cell cycle regulation, stress response, differentiation, apoptosis, and invasion, and over the past decade they have been found to have key roles in cancers [13–15], including lung cancer [16]. Moreover, recent studies have suggested a link between expression of some miRNAs and radiotherapy, particularly in lung cancer [17–19].

microRNA-21 (miR-21) is a miRNA which has been reported to be overexpressed in many human malignancies including NSCLC [20–22]. Interestingly, miR-21 was found to be upregulated in radiotherapy resistant NSCLC cells relative to radiosensitive counterparts [18]. In addition, Wang et al. also reported that, comparing with radiotherapy resistant NSCLC patients, miR-21 was greatly downregulated in radiotherapy sensitive group [23]. Considering miR-21 as a putative regulator of NSCLC radiotherapy resistance, we explore the role of miR-21 in radiotherapy resistance of NSCLC A549 cells and the potential molecular mechanism in the present study.

## 2. Materials and Methods

### 2.1. Cell Culture

The NSCLC cell line A549 was cultured in Dulbecco's modified Eagle's medium (Invitrogen, Carlsbad, CA) supplemented with 10% fetal bovine serum, 100 U/mL penicillin, and 100 *μ*g/mL streptomycin. Cell cultures were incubated in a humidified atmosphere of 5% CO_2_ at 37°C.

### 2.2. Transfection

Anti-miR-21 (5′-UCAACAUCA-GUCUGAUAAGCUA-3′) and the negative control oligonucleotides (NC, 5′-CAGUACUUUUG-UGUAGUACAA-3′) were obtained from Ambion Inc. (Austin, TX, USA). The transfection was performed using LipofectamineTM 2000 (Invitrogen, USA) according to the instructions provided by the manufacturer. The transfected cells were resuspended and cultured in regular culture medium for 48 h before analysis.

### 2.3. Detection of miR-21 by TaqMan Real-Time PCR

PCR-based detection of miR-21 was performed by the TaqMan miRNA assays (ABI, Forest City, CA) as described previously [24, 25]. The real-time PCR results, recorded as threshold cycle numbers (Ct), were normalized against an internal control (U6 RNA) and then expressed as fold changes [25].

### 2.4. Ionizing Radiation

48 h after anti-miR-21 or anti-miR-NC transfection, subconfluent cell monolayers were treated with *γ*-ray ionizing radiation (IR) from a ^60^Co source (PLA General Hospital, Beijing, China) at a rate of 2.4 Gy/min.

### 2.5. Clonogenic Survival Analysis

After exposure to various doses of IR, cells were trypsinized, washed, and replated at 200 cells per 10-cm dishes. Cells were grown for 14 days, fixed with ethanol, and stained with Giemsa to detect colonies. The number of colonies containing at least 50 cells was determined, and surviving fractions were calculated.

### 2.6. MTT Assay

Twenty-four hours before IR, 200 *μ*L cells were seeded to 96-well microtiter plate at 5 × 10^4^ cells/mL. Three days after IR, 10 *μ*L MTT reagent was added to each well, followed by incubation for 4 h at 37°C. The supernatants were aspirated and the reaction was terminated by adding 100 *μ*L DMSO. The contents of the plates were mixed for 10 min and the absorbance was read at 490 nm. All experiments were performed three times and the average results were calculated.

### 2.7. Flow Cytometry

Attached cells were harvested at 48 h after IR for apoptosis detection using the annexin V-FITC apoptosis detection kit (Sigma, Louis, MO). Briefly, the cells were washed twice with DPBS and then were resuspended in 1× binding buffer at a concentration of 1 × 10^6^ cells/mL. 5 *μ*L of annexin V-FITC conjugate and 10 *μ*L of propidium iodide solution were added to 500 *μ*L of each cell suspension in a plastic 12 mm × 75 mm test tube, followed by incubation at room temperature for 15 min and protection from light. The fluorescence of the cells was determined immediately with a flow cytometer. 10 ng/mL of PI3K activator IGF-1 (Prospec-Tany, Rehovot, Israel) was used in the apoptosis assay.

### 2.8. Western Blot Analysis

Cells were lysed in lysis buffer (20 mM Tris-HCl pH 7.4, 150 mM NaCl, 1% Triton X-100, 0.1 mM EDTA, 1 mM EGTA, 2 mM sodium orthovanadate, 2 mM NaF, and Complete TM Protease Inhibitor Mix [Roche Applied Science, Mannheim, Germany]) for 20 min on ice and cleared by centrifugation at 12,000 rpm and 4°C. Proteins were resolved on a 10% SDS PAGE gel, transferred onto nitrocellulose membranes, and blocked with 5% nonfat dry milk in TBST (10 mM Tris-HCl pH 7.5, 100 mM NaCl, and 0.05% Tween 20), followed by incubation with a primary antibody [total and anti-phosphorylated-Akt (Ser473) antibody (Cell Signaling Biotechnology, Beverly, MA, USA)]. Blots were washed and incubated with horseradish peroxidase-conjugated secondary antibody. Antibody complexes were visualized using an enhanced chemiluminescence-Western blotting detection system (Thermo Fisher Scientific, Inc., Rockford, IL, USA).

### 2.9. Statistical Analysis

Statistical analysis was performed using SPSS 13.0. The results from three independent experiments were presented as the means ± standard deviation. Statistical analyses were done by Student's *t*-test. *P* value < 0.05 was considered statistically significant.

## 3. Results

### 3.1. miR-21 Expression Was Knocked down in A549 Cells by Anti-miR-21 Transfection

**Figure 1:**
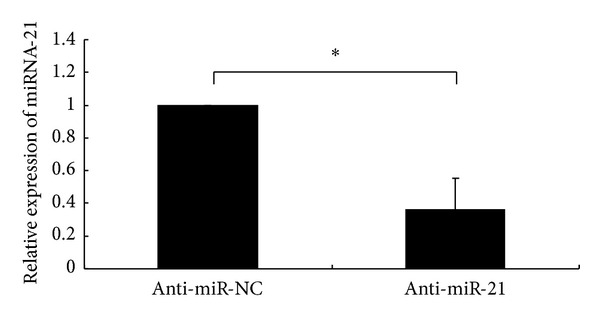
miR-21 expression was knocked down by transfecting NSCLC A549 cells with anti-miR-21. miR-21 expression in A549 cells at 48 h after transfection with anti-miR-NC or anti-miR-21 was detected by TaqMan real-time quantitative RT-PCR. The mean and standard deviation of expression levels relative to U6 expression levels are shown and are normalized to the expression in A549 cells transfected with anti-miR-NC. All experiments were performed at least in triplicate. *P < 0.05 versus cells transfected with anti-miR-NC.

To confirm knockdown efficiency of anti-miR-21 transfection, the relative of miR-21 expression level was detected by real-time quantitative RT-PCR. Compared with anti-miR-NC-transfected A549 cells, the level of miR-21 expression in anti-miR-21-transfected cells was significantly decreased by about 64% (Figure 1).

### 3.2. Downregulation of miR-21 Inhibited Survival Capacity of A549 Cells after IR

**Figure 2:**
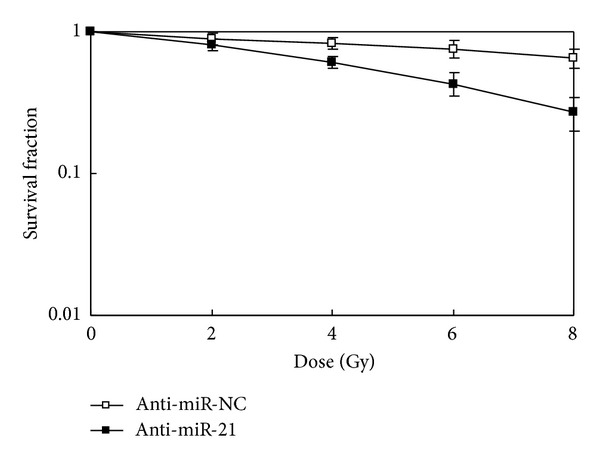
Clonogenic survival of NSCLC A549 cells after varying doses of ionizing radiation. A549 cells were transfected with either anti-miR-21 or anti-miR-NC and 48 h later were irradiated followed by a further incubation for 24 h at 37°C before trypsinization and plating for clonogenic survival. After 14-day incubation, colonies were stained, and the surviving fractions were determined. *P < 0.05 versus cells transfected with anti-miR-NC. Each value represents the means ± SD for three independent experiments.

To assess whether miR-21 downregulation could sensitize NSCLC A549 cells to IR, the A549 cells transfected with either anti-miR-NC or anti-miR-21 were irradiated and their response was analysed. In clonogenic survival analysis, we observed the expected decreased survival capacity of A549 cells transfected with anti-miR-21 14 days after IR (Figure 2). Forty-eight hours after transfection, A549 cells were treated with various doses of IR (0, 2, 4, 6, or 8 Gy) and the survival fractions upon IR were detected. As shown in Figure 2, after IR at 4, 6, or 8 Gy, the survival fraction of A549 cells in anti-miR-21-transfected group (0.61 ± 0.06, 0.43 ± 0.08, and 0.27 ± 0.07, resp.) was significantly lower than that in anti-miR-NC-transfected group (0.83 ± 0.08, 0.76 ± 0.11, and 0.65 ± 0.10, resp.), indicating that downregulation of miR-21 could significantly enhance the sensitivity of A549 cells to IR.

### 3.3. Downregulation of miR-21 Suppressed Proliferation of A549 Cells after IR

**Figure 3:**
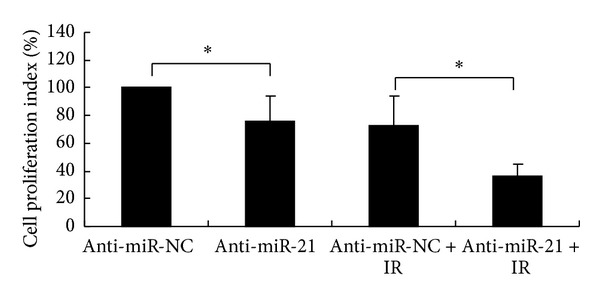
Proliferation of NSCLC A549 cells after ionizing radiation (IR). A549 cells were transfected with either anti-miR-21 or anti-miR-NC and 48 h later were exposed to 8 Gy of IR and the growth characteristics of A549 cells were determined by MTT assay 72 hours after IR. The anti-miR-NC-transfected sample was normalized to 100% cell viability. The data represent the means ± SD of three separate experiments. Student's *t*-test was used to analyze the statistics (*P < 0.05).

To confirm the increased IR sensitivity of A549 cells, the effect of miR-21 on cell proliferation was further analysed at 72 h after IR (Figure 3). Downregulation of miR-21 expression was found to reduce cell proliferation, as demonstrated by the decreased proliferation index of cells transfected with anti-miR-21 compared with anti-miR-NC (75.6 ± 18.96% versus 100%, *P* < 0.05). Importantly, a more pronounced growth inhibition of A549 cells was found when miR-21 was knocked down in combination with IR. This inhibition of cell growth in the combined treatment (anti-miR-21 + IR) was found to be significantly higher compared with that in the sole IR treatment group (proliferation index: 36.1 ± 8.48% versus 73.2 ± 21.37%, *P* < 0.05, Figure 3). This indicates that knockdown of miR-21 sensitizes radioresistant NSCLC A549 cells to radiation.

### 3.4. Downregulation of miR-21 Enhanced Apoptosis of A549 Cells Induced by IR

**Figure 4:**
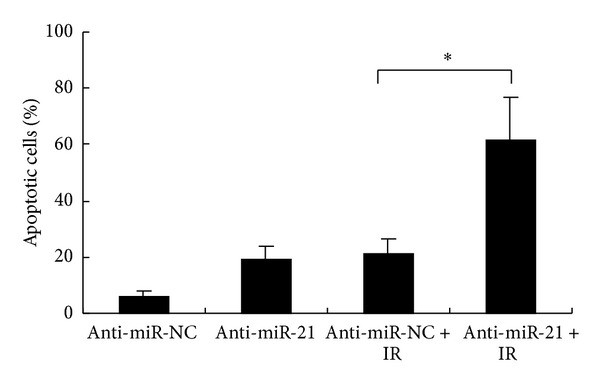
Apoptosis of NSCLC A549 cells after ionizing radiation (IR). Apoptosis in anti-miR-21- (or anti-miR-NC-) transfected A549 cells combined with (or without) IR (8.0 Gy) was detected through annexin V-FITC/PI staining by flow cytometric analysis. The data represent the means ± SD of three separate experiments. Student's *t*-test was used to analyze the statistics (*P < 0.05).

We next explored the role of miR-21 in the apoptosis of NSCLC A549 cells induced by IR. Anti-miR-21 or anti-miR-NC was transfected into A549 cells and was exposed (or sham exposed) to 8 Gy of IR. As shown in Figure 4, the percentage of apoptosis cells in miR-21 knockdown group (anti-miR-21) was significantly higher than that of negative control group (anti-miRNA-NC) at the dose 8 Gy (61.5 ± 15.62 versus 21.2 ± 5.35, *P* < 0.05), indicating that miR-21 knockdown may enhance radiosensitivity of A549 cells by promoting apoptosis and thus confirm a role for miR-21 in the regulation of radiotherapy response of NSCLC.

### 3.5. Downregulation of miR-21 Inactivated PI3K/Akt Signaling Pathway Induced by IR

**Figure 5:**
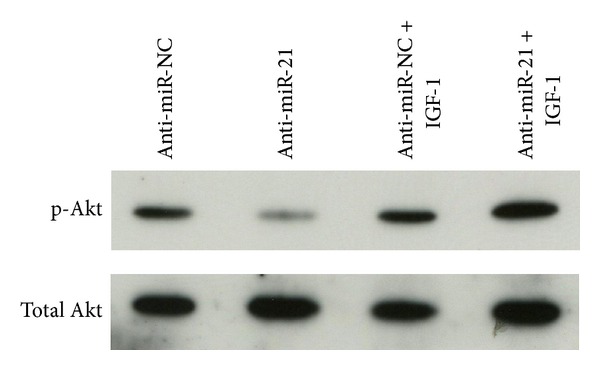
Suppression of ionizing radiation-induced phosphorylated-Akt (p-Akt) upregulation by anti-miR-21 in NSCLC A549 cells. The expression level of the phosphorylated or total Akt in A549 cells transfected with anti-miR-21 or anti-miR-NC for 48 h followed by ionizing radiation (8 Gy) with or without 10 ng/mL PI3K constituent activator IGF-1 was measured by Western blot. Representative of three independent experiments was shown.

Because the PI3K/Akt signaling pathway is associated with apoptosis, we subsequently examined the potential effects of miR-21 on the activation of PI3K/Akt pathways by IR to explore the potential molecular mechanisms. The activation of PI3K/Akt signaling pathways was measured by Akt phosphorylation on Ser473. By Western blot, we found that the endogenous level of phospho-Akt expression (Ser473) in anti-miR-21-transfected A549 cells was downregulated compared to that in anti-miR-NC-transfected A549 cells after IR (Figure 5). Interestingly, phospho-Akt (Ser473) expression was significantly increased in the case of being treated with IGF-1, a PI3K activator, in anti-miR-NC-transfected A549 cells and even in anti-miR-21-transfected A549 cells after IR (Figure 5). This suggested that activation of PI3K/Akt signaling pathway by IR in A549 cells was suppressed by knockdown of miR-21, and the suppression was reversed by PI3K activator IGF-1.

### 3.6. miR-21 Knockdown Caused Promotion on Apoptosis Induced by IR Was Mediated by PI3K/Akt Signaling Pathway

**Figure 6:**
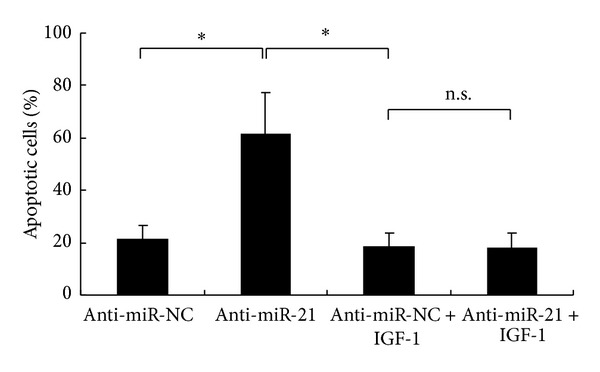
Knockdown of miR-21 promoted NSCLC A549 cells apoptosis via inactivation of PI3K-Akt pathway. Apoptosis induced by 8 Gy ionizing radiation in anti-miR-21- (or anti-miR-NC-) transfected A549 cells combined with (or without) PI3K activator IGF-1 treatment (10 ng/mL) was detected through annexin V-FITC/PI staining by flow cytometric analysis. The data represent the means ± SD of three separate experiments. Student's *t*-test was used to analyze the statistics (*P < 0.05).

To further confirm the molecular mechanisms of radiosensitization by miR-21 knockdown in NSCLC A549 cells, we next treated the cells with or without PI3K activator IGF-1 and then examined the effects of miR-21 downregulation on cell apoptosis induced by IR. As shown in Figure 6, without IGF-1 treatment, the cell apoptosis induced by IR was significantly increased in anti-miR-21-transfected A549 cells (61.5 ± 15.62%) compared with that in anti-miR-NC-transfected A549 cells (21.2 ± 5.35%, *P* < 0.05). However, after activation of PI3K/Akt signaling pathway, the cell apoptosis induced by IR was inhibited in either anti-miR-21-transfected or anti-miR-NC-transfected A549 cells. The percentage of cell apoptosis was not significantly different between these two groups (18.1 ± 5.55% versus 18.3 ± 5.15%, *P* > 0.05). These data showed that, in the condition of PI3K/Akt activation, knockdown of miR-21 did not promote the apoptosis of A549 cells induced by IR, suggesting that PI3K/Akt signaling pathway was the downstream target of miR-21 and the promotive effects of miR-21 knockdown on apoptosis induced by IR were mediated by PI3K/Akt signaling pathway.

## 4. Discussion

It is well known that the acquisition of resistance to radiotherapy, which greatly increases patient morbidity and mortality, is a significant problem in the treatment of NSCLC. Effective treatment which can sensitize the radioresistant NSCLC to radiotherapy is always being sought. Recently, some miRNAs were found to be related to radioresistance. Among them, miR-21 is reported to play a role in radioresistance of cancer, including glioblastoma [26, 27], breast cancer [28], and rectal cancer [29]. But up to now, few researches have studied the correlations between miR-21 expression and radiotherapy sensitivity of NSCLC. Liu et al. reported that miR-21 expression promotes radioresistance in NSCLC, but the related molecular mechanisms were not revealed [30]. The roles of miR-21 in the radiotherapy response of NSCLC are not fully understood and remain to be elucidated. Thus, in the current study, we investigated whether miR-21 could affect the radiosensitivity of NSCLC A549 cells and found that downregulation of miR-21 significantly enhanced the sensitivity of A549 cells to radiotherapy through inhibition of PI3K/Akt signaling pathway.

Our data showed that, following the transfection of anti-miR-21 into A549 cells, the inhibition of survival fraction caused by various doses of IR was enhanced compared with radiotherapy alone. This result suggests that miR-21 is closely associated with the therapeutic efficiency of IR on radioresistant A549 cells and downregulation of miR-21 may sensitize A549 cells to IR.

It is reported that miR-21 could stimulate growth in NSCLC [30, 31]. Accordingly, we also found that the proliferation of A549 cells was inhibited after miR-21 knockdown. Moreover, the inhibition of cell proliferation induced by combination of miR-21 knockdown and IR was more pronounced compared with either miR-21 knockdown or IR treatment, indicating that miR-21 knockdown plays a crucial role in the combined inhibition of cell proliferation and silencing miR-21 may increase the sensitivity of A549 cells to IR.

Cell apoptosis induced by IR is one of the most important effects of tumor radiotherapy. Furthermore, miR-21 is reported to be an antiapoptotic factor in lung cancer [32, 33]. So, we hypothesized that it is possible that miR-21 could affect the apoptosis of NSCLC induced by IR. Our current results demonstrated that miR-21 knockdown promoted apoptosis of A549 cells induced by IR, indicating that the expression of miR-21 could affect radiosensitivity of NSCLC cells, which might be associated with inhibition of apoptosis. This is also in agreement with the previous report [30].

To explore the potential molecular mechanisms of radiosensitization by miR-21 knockdown in NSCLC A549 cells, we focused on analysis of PI3K/Akt signaling pathway because the influence of PI3K/Akt signaling pathway on IR-induced apoptotic propensity is well documented [34, 35]. We examined whether downregulation of miR-21 could affect Akt phosphorylation at Ser473 and/or its total expression and found that miR-21 knockdown suppressed the activation of PI3K/Akt signaling pathway by IR in A549 cells. In addition, the apoptosis induced by IR was enhanced in A549 cells after miR-21 knockdown. This data indicates that the stimulative effects of miR-21 knockdown on A549 cell apoptosis induced by IR are related to the inactivation of PI3K/Akt signaling pathway. Furthermore, with the treatment of PI3K activator IGF-1, we found that the apoptosis of A549 cells induced by IR was not promoted even if miR-21 was downregulated. Our results suggest that the promotive effects of miR-21 knockdown on A549 cell apoptosis induced by IR depend on the inactivation of PI3K/Akt signaling pathway. In the current study, how miR-21 interplays with PI3K/Akt signaling pathway under our experimental conditions is not clear. However, it is reported that molecules such as PTEN have been proposed to be involved in NSCLC cells' radioresistance [36, 37] and miR-21 is related to PTEN with high possibility [30, 38]. In addition, since PTEN, PI3K, and Akt are closely related, it is one of the possible mechanisms that PTEN may play a role in PI3K/Akt signaling pathway mediated radiosensitization of A549 cells by miR-21 knockdown but this still needs further comfirmation in future studies.

In summary, the present study found that downregulation of miRNA-21 sensitized radioresistant NSCLC A549 cells to IR by inhibiting cell proliferation and enhancing apoptosis through inhibition of PI3K/Akt signaling pathway. This information may be useful to develop new treatments for the clinical therapy of NSCLC patients. Further analysis on targets of miR-21 is still of considerable interest, as they may reveal novel radiotherapy sensitization strategies for radioresistant NSCLC.
